# Intermittent Arterial Hemorrhage From Ureteral‐Arterial Fistula After Long‐Term Ureteral Stenting

**DOI:** 10.1155/criu/1581782

**Published:** 2026-04-25

**Authors:** Kentaro Yoshihara, Kojiro Tashiro, Maria Okamoto, Keiichiro Miyajima, Ryoko Sakakibara, Kensuke Fujiwara, Yusuke Takahashi, Masaki Hashimoto, Akira Hisakane, Fumihiko Urabe, Hiroki Yamada, Takahiro Kimura

**Affiliations:** ^1^ Department of Urology, The Jikei University School of Medicine Katsushika Medical Center, Tokyo, Japan; ^2^ Department of Urology, The Jikei University School of Medicine, Tokyo, Japan, jikei.ac.jp

**Keywords:** long-term stenting, ureteral arterial fistula, ureteral stent

## Abstract

Ureteroarterial fistula (UAF) is a rare but potentially life‐threatening condition that can cause massive hematuria. We report a case of UAF in a 74‐year‐old woman with a history of pelvic surgery, radiotherapy, and long‐term ureteral stenting who presented with severe intermittent hematuria. The diagnosis was challenging because initial contrast‐enhanced computed tomography showed no findings suggestive of UAF, and the bleeding pattern led to an initial suspicion of radiation cystitis. As hemorrhage recurred and anemia progressed, angiography was eventually performed and revealed a pseudoaneurysm of the left external iliac artery, leading to the diagnosis of UAF. Endovascular stent‐graft placement achieved successful hemostasis. This case highlights that UAF should be suspected early in high‐risk patients with intermittent hematuria, even when initial imaging findings are negative.

## 1. Introduction

Ureteral stent placement is a common urological procedure used to manage ureteral stenosis caused by malignancy, postoperative stricture, or occlusion. In certain patients, stenting becomes a long‐term intervention requiring periodic replacement. Long‐term stenting is associated with complications such as catheter obstruction, technical difficulties during stent exchange, and, rarely, ureteroarterial fistula (UAF). UAF is a potentially fatal condition in which arterial blood enters the ureter, resulting in life‐threatening hemorrhage. We present a case of UAF in a patient with long‐term periodic ureteral stent replacement who developed intermittent severe hematuria despite no suggestive findings on contrast‐enhanced computed tomography (CT).

## 2. Case Presentation

**Figure 1:**
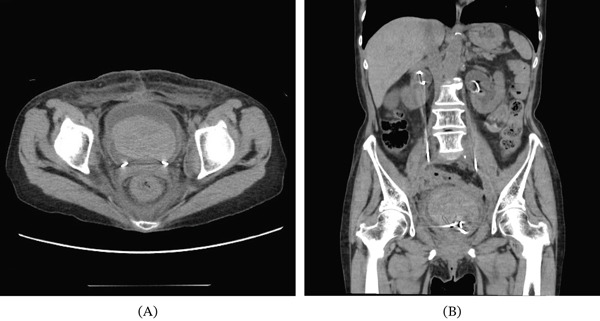
Noncontrast CT images on admission. (A) Intravesical hematoma. (B) No hematoma in the renal pelvis

**Figure 2:**
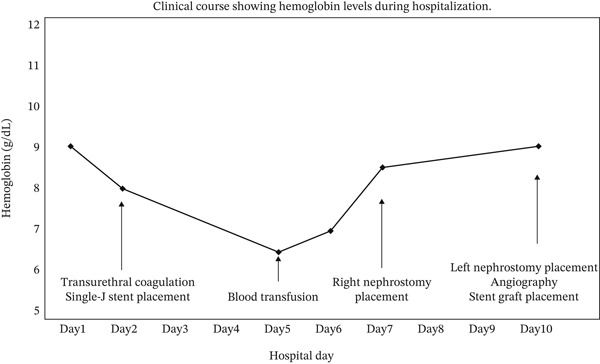
Clinical course showing hemoglobin levels during hospitalization.

**Figure 3:**
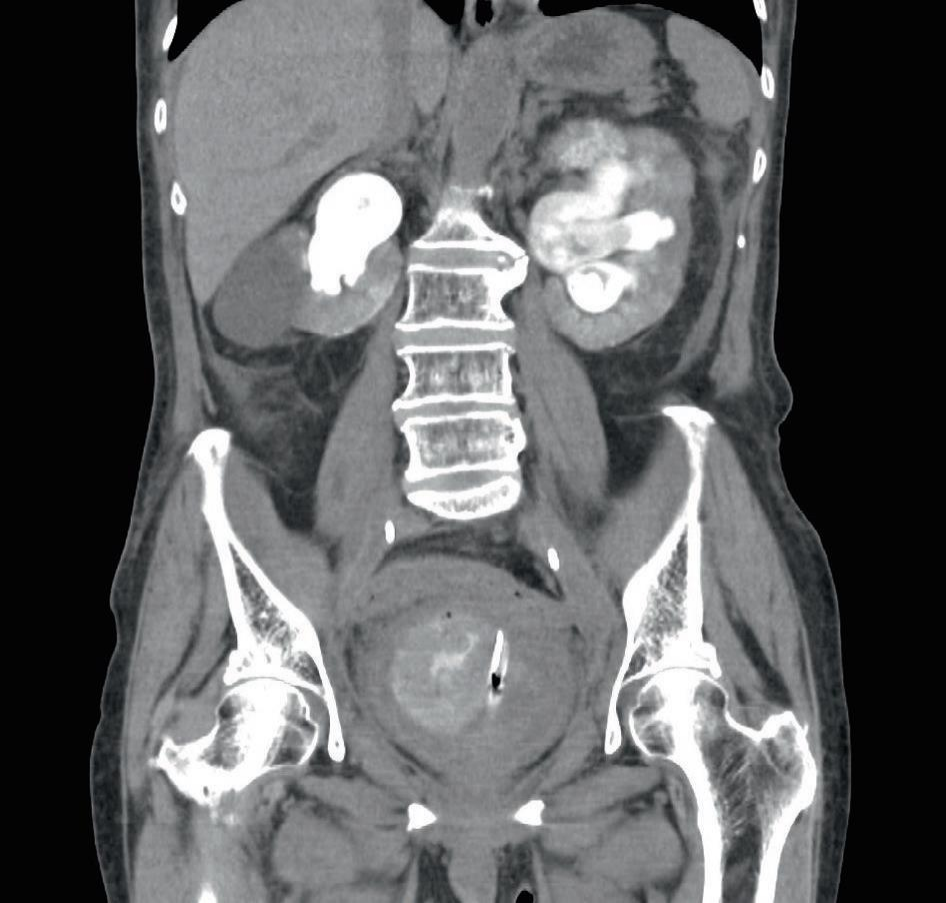
Contrast CT image after intermittent bleeding. A hematoma filling the left upper tract, with no evidence of arterial hemorrhage.

A 74‐year‐old woman with a history of cervical cancer underwent radical hysterectomy with bilateral salpingo‐oophorectomy and lymph node dissection, followed by adjuvant chemotherapy and pelvic radiotherapy. One year later, postoperative changes led to bilateral ureteral stenosis of 2 cm at the U2 level. Bilateral double‐J ureteral stents were then inserted. The following year, these were replaced with bilateral metallic stents, which were exchanged at 6‐month intervals. Two years after the initial stent placement, the patient presented with severe gross hematuria. On admission, CT demonstrated a large intravesical hematoma (Figure 1A) without evidence of hematoma in the renal pelvis or upper urinary tract (Figure 1B). Based on these findings, hemorrhage secondary to radiation cystitis was suspected. Although UAF was a potential differential diagnosis because of the patient′s history of pelvic radiotherapy and long‐term ureteral stenting, it was not strongly suspected at this stage. This was because the initial CT findings appeared more consistent with bladder bleeding, and there were no clear findings suggestive of arterial hemorrhage from the upper urinary tract. Because of persistent bleeding, transurethral coagulation was performed on hospital Day 2. Cystoscopy revealed mild radiation cystitis and bleeding from the left ureteral orifice. Because the bleeding was not clearly pulsatile on endoscopic examination, venous bleeding was considered more likely than arterial bleeding at that time. Therefore, a left single‐J ureteral stent was placed to maintain access to the left upper tract. Although this intervention temporarily achieved hemostasis, hematuria recurred, requiring repeated blood transfusions on hospital Day 5. Hemoglobin levels gradually declined during the clinical course, as shown in Figure 2. Because anemia persisted, follow‐up contrast‐enhanced CT demonstrated a hematoma occupying the left upper urinary tract without evidence of arterial extravasation (Figure 3). Intermittent bleeding episodes resulted in urinary retention and right hydronephrosis. Consequently, the right double‐J ureteral stent was removed and replaced with a percutaneous nephrostomy on hospital Day 7. Idiopathic renal hemorrhage from the left kidney was considered, and after discussion with the patient and her family, conservative management was elected.

**Figure 4:**
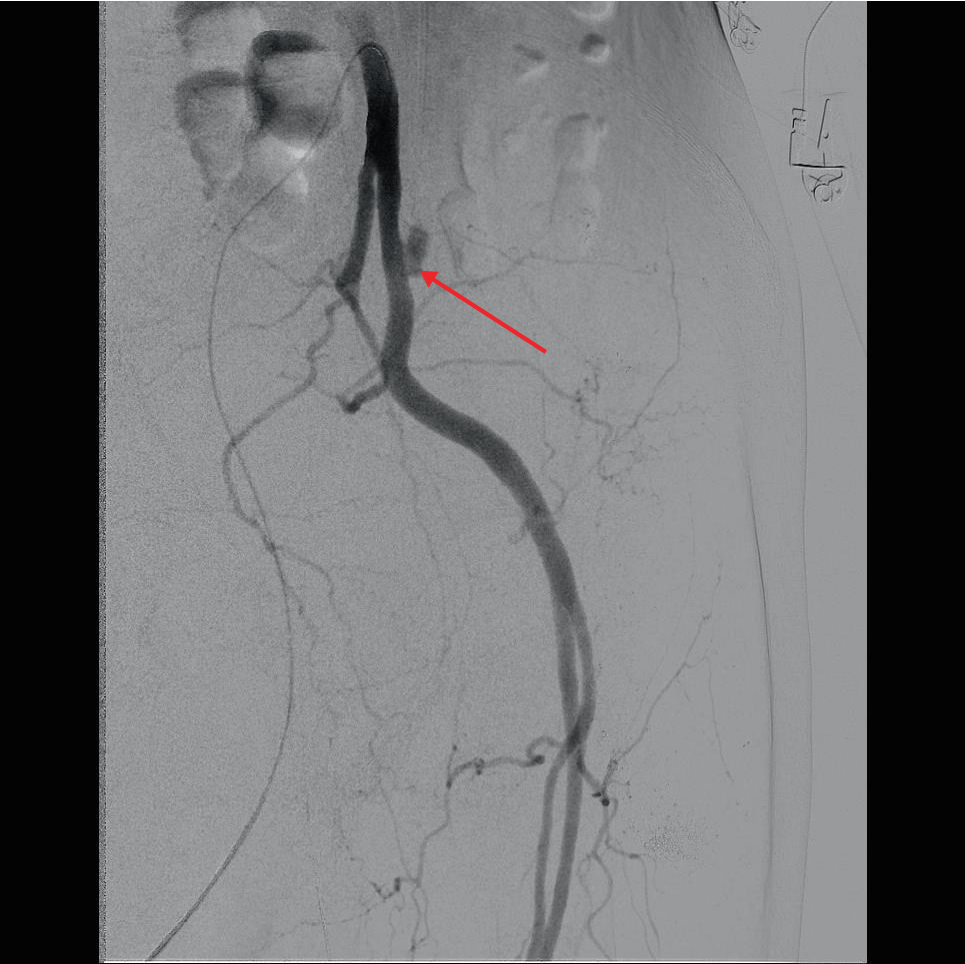
An emergency angiogram on Day 10. Ureteral‐arterial fistula with pseudoaneurysm in the external iliac artery (red arrow).

On Day 10 of admission, the patient developed significant anemia due to ongoing intermittent bleeding. A left percutaneous nephrostomy was placed to provide direct access to the left upper tract, and hemostasis was attempted with a nephroscope. During the procedure, profuse hematuria was observed through the urethral catheter, and the patient became hypotensive and developed shock. Because the clinical course suggested arterial hemorrhage, the left common iliac angiography was performed, and digital subtraction angiography (DSA) demonstrated a pseudoaneurysm in the proximal left external iliac artery (Figure 4). After confirming the origin of the internal iliac artery, a 7‐mm × 5‐cm Viabahn stent graft was deployed with the segment just distal to the bifurcation used as the proximal landing zone. Postdeployment DSA confirmed the disappearance of the pseudoaneurysm. Additional postdilatation was then performed with a 7‐mm × 4‐cm PTA balloon. Hematuria resolved following the intervention, and the patient was discharged after rehabilitation.

## 3. Discussion

UAF represents an abnormal communication between the ureter and the iliac artery or aorta. The hallmark clinical manifestation is gross hematuria. Although rare, UAF is often life‐threatening and requires timely recognition and prompt intervention [1].

Well‐established risk factors include prior pelvic surgery, radiation therapy, and prolonged ureteral catheterization, all of which were present in the current case. Enhanced CT detects UAF in approximately 50% of cases, whereas retrograde pyelography (45%–60%) and angiography (23%–41%) may yield false‐negative results [2].

Several studies have emphasized that enhanced CT may fail to detect UAF when the fistula is transiently occluded by a stent or thrombus [3, 4]. In CT‐negative cases, provocative angiography during stent removal can be diagnostic; however, this procedure carries a high risk of massive hemorrhage and requires preparation for immediate endovascular intervention [5].

In the present case, the patient had multiple established risk factors for UAF, including prior pelvic surgery, pelvic radiotherapy, and prolonged ureteral stenting. In retrospect, UAF should have been considered earlier in the diagnostic process. However, the initial CT demonstrated a large intravesical hematoma without evidence of upper urinary tract hematoma, and cystoscopy showed no clearly pulsatile bleeding from the ureteral orifice. These findings contributed to the initial suspicion of radiation cystitis and delayed the diagnosis of UAF. Previous studies have indicated that approximately 99% of patients present with gross hematuria, with 14% experiencing intermittent massive bleeding [6, 7]. In addition, UAF may be associated with back pain and hydronephrosis and, in rare instances without hematuria, it may manifest as peripheral vascular symptoms, such as lower limb ischemia [8]. These observations emphasize the importance of integrating medical history and clinical presentation to achieve an early diagnosis of UAF.

The pathogenesis of UAF involves arterial pulsation compressing the ureteral wall between the stent and the arterial wall, resulting in ischemic necrosis and subsequent fistula formation [9]. Metallic stents are highly resistant to external compression and maintain luminal patency in malignant obstruction [10]. However, their increased radial force may impose excessive pressure on the ureteral wall. To date, no studies have compared the risk of UAF between metallic and polymer stents, and the contribution of metallic stents to fistula formation remains hypothetical. Nevertheless, in patients with established risk factors such as prior pelvic surgery, the presence of a metallic stent may increase clinical suspicion for UAF. Management options for UAF include open surgery, arterial embolization, and endovascular therapy. Historically, open surgical repair, consisting of nephroureterectomy combined with arterial bypass, was considered the standard approach [11]. Since 2000, however, endovascular therapy has emerged as the preferred treatment modality. Although endovascular strategies include coil embolization and covered stent‐graft placement, the latter may be complicated by rebleeding, lower limb ischemia, or infection. Therefore, careful long‐term follow‐up is essential to detect and manage potential complications [11].

In our study, bilateral nephrostomies were maintained without ureteral stenting to prevent recurrence of UAF. At 1‐year follow‐up after endovascular stenting, no complications such as rebleeding or infection were observed.

In conclusion, we report a case of UAF presenting with intermittent severe hematuria, which made the diagnosis challenging. In patients with established risk factors such as prior pelvic surgery, pelvic radiotherapy, and prolonged ureteral stenting, UAF should be suspected early even when initial imaging findings are negative. Early angiographic evaluation should be considered in such high‐risk settings.

**Nomenclature**
UAF: ureteroarterial fistula
CT: computed tomography
DSA: digital subtraction angiography

## Funding

No funding was received for this manuscript.

## Consent

Although there is no patient‐identifiable data included in this case report, written informed consent was obtained from the patient for publication.

## Conflicts of Interest

The authors declare no conflicts of interest.

## Data Availability

The data that support the findings of this study are available on request from the corresponding author. The data are not publicly available due to privacy or ethical restrictions.
